# Reduction in MLKL-mediated endosomal trafficking enhances the TRAIL-DR4/5 signal to increase cancer cell death

**DOI:** 10.1038/s41419-020-02941-9

**Published:** 2020-09-11

**Authors:** Se-Yeon Park, Han-Hee Park, Sang-Yeong Park, Sun Mi Hong, Seongmin Yoon, Michael J. Morgan, You-Sun Kim

**Affiliations:** 1 Department of Biochemistry, School of Medicine, Ajou University, Suwon, Korea; 2 Department of Biomedical Sciences, Graduate School, Ajou University, Suwon, Korea; 3 Department of Biomolecular Sciences, The Weizmann Institute of Science, 76100 Rehovot, Israel; 4 Department of Natural Sciences, Northeastern State University, Tahlequah, OK 74464 USA

**Keywords:** Targeted therapies, Necroptosis

## Abstract

Mixed lineage kinase domain-like (MLKL) is an essential molecule of necroptosis, a cell death process that is initiated by direct disruption of the plasma membrane. During necroptosis, MLKL is phosphorylated by receptor interacting protein kinase-3 (RIPK3 or RIP3), and then translocates to the plasma membrane to disrupt membrane integrity. Recent data suggest that MLKL also has a RIP3-indendent function in the generation of intraluminal and extracellular vesicles (EVs), as well as in myelin sheath breakdown when promoting sciatic nerve regeneration. Here we show that depletion of MLKL enhances TRAIL-induced cell death in a RIP3-independent manner. Depletion of MLKL leads to prolonged cytotoxic signals that increase TRAIL-induced cell death. Initially, TRAIL binds to DR5 at the cell surface and is endocytosed at similar rates in MLKL-expressing and MLKL-depleted cells, eventual degradation of intracellular TRAIL by the lysosome is delayed in MLKL-depleted cells, corresponding with prolonged/enhanced intracellular signals such as p-ERK and p-p38 in these cells. Colocalization of TRAIL with the marker of early endosomes, EEA1 suggests that TRAIL is accumulated in early endosomes in MLKL-depleted cells compared to MLKL-expressing cells. This indicates that depletion of MLKL reduces receptor-ligand endosomal trafficking leading to increased TRAIL-cytotoxicity. An MLKL mutant that compromises its necroptotic function and its function in the generation of EVs was sufficient to rescue MLKL deficiency, suggesting that the N-terminal structural elements necessary for these functions are not required for the function of MLKL in the intracellular trafficking associated with regulating death receptor cytotoxicity. A reduction in MLKL expression in cancer cells would therefore be expected to result in enhanced TRAIL-induced therapeutic efficacy.

## Introduction

Mixed lineage kinase domain-like (MLKL) has been identified as a key molecule of necroptosis^1–3^. MLKL is required to form the necrosome complex with receptor-interacting protein kinases 1 and 3 (RIPK1 or RIP1;RIPK3 or RIP3), and RIP3-dependent plasma membrane localization of MLKL is necessary for programmed necrotic cell death to occur^4–6^. The translocation of MLKL to the membrane has been alternately reported to lead to plasma membrane disruption by through its activation of ion channels or by directly permeabilizing the plasma membrane, perhaps through binding to phosphatidylinositol phosphates^7,8^.

Recent data suggest that MLKL has alternative functions that occur independent of RIP3. Yoon et al., reported that MLKL affected endosomal transport by promoting the generation of intraluminal and extracellular vesicles, a function which was independent of RIP3^9^. They observed that MLKL depletion reduced the rates of intracellular degradation of some ligands and their receptors and suggested that this was due to decreased MLKL-dependent endosomal trafficking. A RIP3-independent contribution of MLKL was also observed in injury-induced activation signals^10^. In this case, sciatic nerve injury was shown to induce increased MLKL expression in Schwann cells and this promoted myelin sheath breakdown to furthers sciatic nerve regeneration in the absence of RIP3.

TRAIL (TNF-related apoptosis-inducing ligand) is a member of the TNF superfamily and it initiates apoptosis selectively in cancer cells, while having limited effect on normal cells^11,12^. TRAIL binding to its death receptors (DR4 and DR5, also referred to as TRAIL-R1 and TRAIL-R2) induces the formation of the death-inducing signaling complex (DISC) by the recruitment of the adaptor Fas-associated death domain (FADD) and the initiator caspase-8^13,14^. In addition to initiating cell death, binding of TRAIL to its apoptosis-inducing death receptors (DR4 and DR5) stimulates their internalization via clathrin-mediated endocytosis (CME)^15,16^. Receptor-mediated endocytosis plays a critical role in regulating signaling^17,18^, by either promoting endocytosis of ligand-receptor complexes and attenuating cell-surface signaling, or by promoting the formation of endosomes that can serve as signaling platforms for these complexes^17,19^. TRAIL receptor internalization occurs by a clathrin-dependent mechanism. TRAIL-activated DRs trigger the ryanodine receptor (RyR)-dependent release of Ca^2+^ from endoplasmic reticulum (ER) stores, subsequent calcineurin-dependent dephosphorylation, and consequent activation of Dyn1, leading to ligand and receptor-selective uptake of DRs and the attenuation of their apoptotic signaling^20^.

In this study, we investigated the effects of the endosomal trafficking functions of MLKL on TRAIL-induced cell death in cancer cells. We found that depletion of MLKL enhances TRAIL and TNFα-induced apoptosis in various cancer cell lines and with the enhancement of cell death being RIP3-independent. Slowdown of degradation of DR5 by MLKL silencing and enhanced co-staining of internalized TRAIL with EEA1, suggesting buildup of TRAIL in the early endosomes, indicates that MLKL typically functions to promote endosomal trafficking and, therefore, eventual lysosomal degradation. Depletion of MLKL, therefore, results in the enhancement of TRAIL-induced apoptosis through defects in trafficking of TRAIL/TRAIL receptor complexes. Depletion of MLKL also prolongs other TRAIL-induced signaling events suggesting that MLKL facilitates endosomal trafficking of TRAIL receptor complexes, thus promoting their degradation.

## Materials and methods

### GST-TRAIL purification

#### Extraction, purification of GST-TRAIL

A GST-TRAIL expression plasmid was transformed in BL21. The selected colonies were incubated in LB with ampicillin at 37 °C. And then 0.4 mM Isopropyl β-D-1-thiogalactopyranoside (IPTG) was added and the culture was incubated for 5 more hours at 37 °C. Bacteria pellets were then collected by ultracentrifuge. Pellets were lysed in lysis buffer (50 mM Tris-HCl (pH 7.4), 50 mM NaCl, 5 mM EDTA, 1 mM DTT, 0.2% Triton X-100, 1 mM EGTA, 0.1 mg/ml lysozyme, 1 mM PMSF, protein inhibitor in H_2_O), sonicated, 10% Triton-X-100 was added and the lysates were further incubated for 2 h at 4 °C. Lysates were centrifuged at 12,000 rpm for 10 min at 4 °C. Supernatants were incubated with glutathione sepharose beads (GE Healthcare, 17-0756-01) for 4 h at 4 °C. Beads were then collected and washed 3 times in wash buffer I (50 mM Tris-HCl (pH 7.4), 500 mM NaCl, 1 mM EGTA, 10% glycerol, 0.1% Triton X-100, 0.1% β*-*mercaptoethanol, 1 mM PMSF in H_2_O*)*. This was repeated once with wash buffer II (50 mM HEPES (pH 7.5), 100 mM NaCl, 1 mM EGTA, 10% glycerol in H_2_O). The GST-tagged proteins were eluted with elution buffer (50 mM HEPES (pH 7.5), 100 mM NaCl, 10% glycerol, 40 mM L-glutathione reduced, 0.03% Triton X-100 in H_2_O).

#### Dialysis

Dialysis membrane (Spectra/Por dialysis membrane; REPLIGEN) was activated in boiling water for 5 min and allowed to cool. The purified GST-TRAIL was put into an activated membrane and dialyzed in dialysis buffer (20 mM Tris-HCl (pH7.5), 20% glycerol, 100 mM KCl, 0.2 mM EDTA, 2 mM DTT, 10 mM β-glycerol phosphate in H_2_O) for overnight at 4 °C. After dialysis, the purity and quantity of GST-TRAIL by gel electrophoresis and Coomassie blue staining and comparing the bands to known amounts of Bovine Serum Albumin on the same gel.

### Antibodies and chemical reagents

Antibodies used in immunoblotting and immunofluorescence: anti-MLKL (Abcam, ab184718, 1:2000), anti-RIP3 (Cell Signaling Technology, 13526, 1:1000), anti-ACTIN (Santa Cruz Biotechnology, 47778, 1:5000), anti-VINCULIN (Sigma-Aldrich, V9131, 1:5000), anti-PARP (Cell Signaling Technology, 9542, 1:1000), anti-AP2a (BD biosciences, 610501, 1:1000), anti-Caspase-3 (Cell Signaling Technology, 9662, 1:1000), anti-Caspase-8 (Cell Signaling Technology, 9746, 1:1000), anti-FADD (BD biosciences, 610400, 1:1000), anti-DR5 (Abcam, ab199357, 1:1000), anti-EGFR (Abcam, ab2430, 1:1000), anti-p-ERK (Cell Signaling Technology, 9101, 1:1000), anti-p-AKT (Cell Signaling Technology, 9271, 1:1000), anti-p-p38 (Cell Signaling Technology, 9215, 1:1000) and anti-GST (Abcam, ab9085, 1:500). TNF-α and zVAD were purchased from R&D Systems. Etoposide and Dynasore were purchased from Sigma-Aldrich. SMAC mimetic (LCL-161) was purchased from Adooq Bioscience. 4 hydroxytamoxifen was purchased from Sigma-Aldrich.

### Cell culture

HeLa, HeLa (RIP3), HT-29 and HEK293T cells were grown in Dulbecco’s Modified Eagle’s Medium (DMEM) supplemented with 10% fetal bovine serum (FBS). H2009, HCC4006, MDA-MB-231 and A549 cells were grown in Roswell Park Memorial Institute (RPMI) 1640 media supplemented with 10% FBS. To generate cell lines stably expressing the RIP3 construct, HeLa cells were infected with pLX303-hRIP3 lentivirus. The MLKL mutant was expressed inducibly in HT-29 cells and the cells were induced to express MLKL by their treatment with 4-hydroxytamoxifen (1 μM) for indicated durations^9^.

### Lentiviral shRNA experiments

MISSION short-hairpin RNA (shRNA) plasmids targeting hMLKL mRNA (NM_152649), and non-targeting control sequences (NM_027088) were obtained from Sigma-Aldrich. Lentiviral plasmids were transfected into 293T cells (System Biosciences, LV900A-1) using Lipofectamine 2000 (Invitrogen, 11668019). Pseudoviral particles were collected 48 h after the lentiviral plasmid transfection and infected into cells with polybrene (8 μg/mL). Infected cells were puromycin selected two days after infection, and knockdown was confirmed by immunoblotting.

### Cytotoxicity assay

Representative images were taken by a phase-contrast microscope. Cell viability was determined using tetrazolium dye colorimetric tests (MTT assay) read at 570 nm. The mean ± STDEV of duplicates is presented.

### Immunoblot analysis

Cells were lysed in M2 buffer containing 20 mM Tris-HCl (pH 7.6), 0.5% NP-40, 250 mM NaCl, 3 mM EDTA, 3 mM EGTA, 2 mM DTT, 0.5 mM PMSF, 20 mM β-glycerol phosphate, 1 mM sodium vanadate, and 1 μg/ml leupeptin. Equal amounts of cell extracts were resolved by SDS-PAGE and analyzed by immunoblotting.

### Ligands and receptor uptake assays, and Immunofluorescence staining

#### Ligands and receptor uptake assays

Before ligand treatment, the cells were incubated for 12 h in serum-free media. Cells were treated with GST-TRAIL or EGF for 30 min on ice in CO_2_-independent media (Thermo Fisher Scientific, 18045-088), and rinsed in cold Dulbecco’s phosphate-buffered saline (DPBS); pre-warmed media (37 °C) was added and incubated for the indicated time at 37 °C.

#### Immunofluorescence staining

After the indicated incubation time, cells were fixed in 4% paraformaldehyde for 10 min. Cells were permeabilized with 0.1% Triton X-100 for 15 min. After incubation in a blocking buffer (10% fetal bovine serum in DPBS) for 30 min, the primary antibody EEA1 (BD biosciences, 610457, 1:1000) and anti-GST (Abcam, 9085, 1:1000) was incubated overnight at 4 °C and then FITC-conjugated secondary antibody (Invitrogen, A11008, 1:250) was incubated for 1 h at room temperature. A mounting medium containing DAPI (Vector Laboratories, 94010) was used for counterstaining.

### Flow cytometry analysis

To stain DR5, the cells were harvested and centrifuged at 1000 rpm for 3 min at 4 °C. The cells were rinsed in FACS buffer (0.5% BSA in DPBS) 3 times. A FITC conjugated DR5 antibody (Abnova, MAB4505) was added and incubated for 1 h on ice; cells were then rinsed in FACS buffer at 1000 rpm for 3 min at 4 °C. The cells were resuspended of FACS buffer and analyzed on a flow cytometer. FACS analysis for cell death was down with Annexin V single staining or Annexin V/PI double staining using FITC Annexin V reagent (BD biosciences, 556547) following manufacturer’s instructions.

### GST-pull down assay

Cells were treated with purified recombinant GST-TRAIL and total cell extracts were prepared in M2 lysis buffer and proteins were immobilized on glutathione-sepharose beads (GE Healthcare Life Sciences, 17-0756-01) for 3 h at 4 °C. The beads were then washed extensively with M2 buffer and the bound proteins were analyzed by western blotting.

### Reverse transcription-PCR

Total RNA was extracted using the TRIzol reagent (Life Technologies, 15596018), according to the manufacturer’s instructions. Using MMLV reverse transcriptase (MGmed, MR10601), cDNA was generated from 1 μg of total RNA for each sample. Equal amounts of cDNA product were employed in reverse transcription-PCR conducted using the GoTaq® Green Master Mix (Promega, M7123). Amplification was executed using the following primers: DR5 forward (5′-GCCTCATGGACAATGAGATAAAGGTGGCT-3′), DR5 reverse (5′-CCAAATCTCAAAGTACGCACAAACGG-3′), beta-ACTIN forward (5′-GTGGGGCGCCCCAGGCA-3′), beta-ACTIN reverse (5′-CTCCTTAAT GTCACGCACGAT-3′).

### Statistical analysis

Data are expressed as the mean ± standard error. Statistical analysis was performed using ANOVA and an unpaired Student′s *t*-test. A *P*-value of 0.05 or less was considered statistically significant. Statistical calculations were performed using SPSS software for Windows Version 12.0 (SPSS, Chicago, IL).

## Results

### Depletion of MLKL accelerates TRAIL-induced cancer cell death

**Fig. 1:**
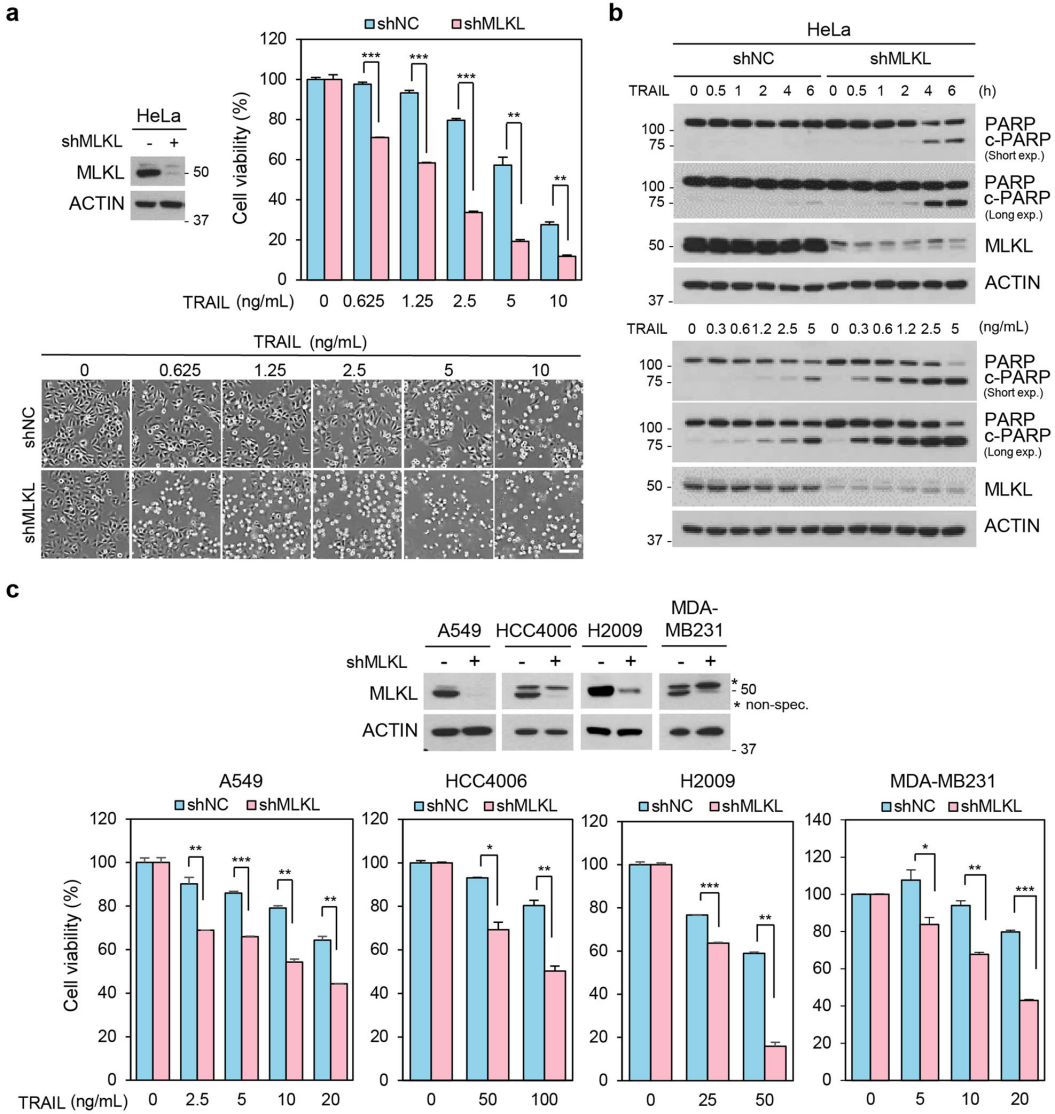
Depletion of MLKL accelerates TRAIL-induced cell death. **a** HeLa cells expressing MLKL shRNA, or a non-silencing control were analyzed by western blotting, and these cells were treated with varying doses of TRAIL for 12 h and cell viability was analyzed by MTT assay (upper panel) or phase-contrast microscopy (bottom panel). The results are presented as means ± SEM. *P < 0.05, **P < 0.01, ***P < 0.001. Scale bars, 100 μm. **b** HeLa cells stably expressing MLKL shRNA or non-silencing control were treated with TRAIL (0.6 ng/ml) in a time-dependent manner (upper panel), and these cells treated for 5 h in dose-dependent manner (bottom panel). The cells were harvested, and total lysates were analyzed by western blotting. **c** A549, HCC4006, H2009, and MDA-MB231 cells expressing MLKL shRNA, or non-silencing control were analyzed by western blotting (upper panel), and these cells were treated with varying doses of TRAIL for 24 h and cell viability was analyzed by MTT assay (bottom panel). The results are presented as means ± SEM. *P < 0.05, **P < 0.01, ***P < 0.001.

MLKL has been reported as a downstream molecule in the RIP3-mediated necroptosis pathway but recently it has been reported that MLKL serves a function independent from cell death in that it associates with the endosomes and promotes endosomal trafficking. Depletion of MLKL therefore reduces effective generation of intraluminal and extracellular vesicles^9^. TRAIL-induced cancer cell death is known to be attenuated by endocytosis^20^. We, therefore, investigated the endosomal trafficking function of MLKL on TRAIL-induced cell death in cancer cells. To test this, we employed HeLa cells depleted of MLKL by RNA interference and treated these cells with different concentrations of TRAIL. Importantly, MLKL depleted cells displayed decreased survival compared with control cells as measured by MTT assay or phase-contrast microscopy (Fig. 1a and Supplementary Fig. 1a) and further confirmed by western blots showing increased PARP and caspase 3 cleavage in the MLKL deficient cells in dose- and time-dependent manner (Fig. 1b and Supplementary Fig. 1b). Consistent with this, various cancer cell lines, including the A549, HCC4006, H2009 (lung cancer cell lines) and MDA-MB231 (breast cancer cell line) cells, showed increased sensitization to TRAIL-induced cell death upon depletion of MLKL (Fig. 1c). To determine whether MLKL overexpression had any effect on TRAIL sensitivity, we established stable HeLa cells expressing high levels of MLKL. Overexpression of MLKL did not affect TRAIL-induced cytotoxicity suggesting that the amount of MLKL expression is not correlated with the rate of cell death but that a certain threshold amount of MLKL expression is sufficient to reduce TRAIL cytotoxicity (Supplementary Fig. 1c). MLKL depletion not only potentiated TRAIL-induced cell cytotoxicity, but also cell death induced by TNFα in combination with SMAC mimetic (Supplementary Fig. 2a). This finding suggests that multiple ligand-receptor complex interactions and/or endosomal trafficking events are being regulated by MLKL.

### RIP3-independent enhancement of cell death by MLKL depletion in TRAIL-mediated cytotoxicity

**Fig. 2:**
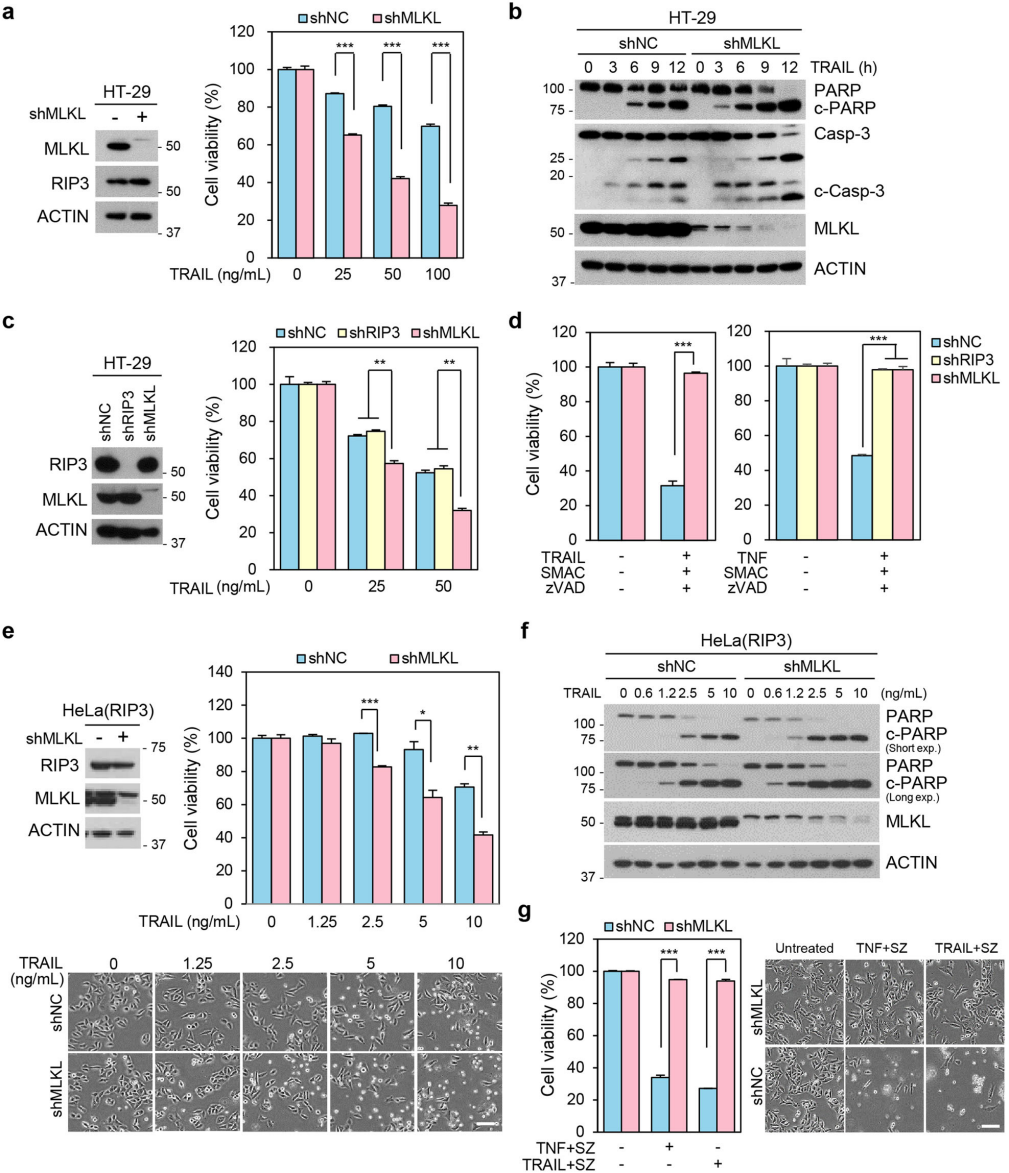
RIP3-independent sensitization of TRAIL-induced cell death by MLKL depletion. **a** HT-29 cells expressing MLKL shRNA, or non-silencing control were analyzed by western blotting (left panel). These cells were treated with varying doses of TRAIL for 48 h and cell viability was analyzed by MTT assay (right panel). The results are presented as means ± SEM. *P < 0.05, **P < 0.01, ***P < 0.001. **b** HT-29 cells stably expressing MLKL shRNA or a non-silencing control were treated with TRAIL (50 ng/ml) in time-dependent manner. The cells were harvested, and total lysates were analyzed by western blotting. **c** HT-29 cells expressing RIP3 shRNA, MLKL shRNA, or a non-silencing control were analyzed by western blotting (left panel), and these cells were treated with varying doses of TRAIL for 48 h. Cell viability was analyzed by MTT assay (right panel). The results are presented as means ± SEM. *P < 0.05, **P < 0.01, ***P < 0.001. **d** HT-29 cells stably expressing MLKL shRNA or a non-silencing control were treated with TRAIL (20 ng/ml) + SMAC mimetic (200 nM) + zVAD (20 μM) (left panel). HT-29 cells stably expressing RIP3 shRNA, MLKL shRNA or non-silencing control were treated with TNF (30 ng/ml) + SMAC mimetic (200 nM) + zVAD (20 μM) (right panel) for 24 h. Cell viability was analyzed by MTT assay. The results are presented as mean ± SEM. *p < 0.05, **p < 0.01, ***p < 0.001. **e** RIP3-expressing HeLa cells expressing MLKL shRNA, or a non-silencing control were analyzed by western blotting, and these cells were treated with varying doses of TRAIL for 12 h and cell viability was analyzed by MTT assay (upper panel) or phase-contrast microscopy (bottom panel). The results are presented as means ± SEM. *P < 0.05, **P < 0.01, ***P < 0.001. Scale bars, 100 μm. **f** RIP3-expressing HeLa cells expressing MLKL shRNA, or a non-silencing control were treated with varying doses of TRAIL for 5 h. The cells lysates were analyzed by western blotting. **g** Cells from (**f**) were treated with TNF + or TRAIL + SMAC mimetic + zVAD for 24 h. Cell viability was analyzed by MTT assay (left panel) or phase-contrast microscopy (right penal). The results are presented as means ± SEM. *P < 0.05, **P < 0.01, ***P < 0.001. Scale bars, 100 μm.

We next examined whether MLKL dependency in response to TRAIL is related with the canonical MLKL-mediated necroptosis pathway, which requires a RIP3-dependent activation process. To determine this, we depleted MLKL in RIP3 positive HT-29 cells, which are extensively used in models of RIP3-dependent MLKL-mediated necroptotic cell death initiated by TNFα^21^. Similar to cells lacking RIP3 expression, HT-29 cells lacking MLKL were sensitized to TRAIL-induced cell death (Fig. 2a) and had more abundant PARP/caspase 3 cleavage upon MLKL depletion (Fig. 2b). RIP3-depleted HT-29 cells, on the other hand, did not have increased TRAIL-induced sensitization, unlike the MLKL depleted HT-29 cells (Fig. 2c), indicating that RIP3 does not play a role in TRAIL-induced sensitization by MLKL depletion. Consistent with the known functions of RIP3 and MLKL in necroptosis, however, both TNFα or TRAIL-induced RIP3-dependent necroptosis (in this case a SMAC mimetic was used to engage necroptosis) was completely attenuated in both MLKL- or RIP3-depleted cells (Fig. 2d). Importantly, MLKL depletion potentiated not only TRAIL-induced apoptosis, but TNF-induced apoptosis as well (Supplementary Fig. 2b), suggesting that MLKL normally attenuates apoptotic cell death induced by diverse ligand/receptor interactions.

We further examined MLKL dependency in response to the chemotherapeutic agent, etoposide. Our previous work concluded that DNA damaging agents activate RIP3- and MLKL-dependent necroptosis^22^. MLKL-depletion in this case decreased etoposide cytotoxicity (Supplementary Fig. 2c). MLKL-depletion, however, had neither a positive or negative affect on etoposide cytotoxicity in HeLa cells, which lack RIP3 (Supplementary Fig. 2d). We, therefore, conclude that MLKL can inhibit cell death initiated by death receptor-mediated apoptosis but, as we have previously shown^22^, it promotes cell death in the same cells during necroptosis. As further evidence that this is the case, we repeated apoptotic and necroptotic experiments in HeLa cells that have ectopic RIP3 expression. Consistent with our data in HT-29 cells, depletion of MLKL increased TRAIL-induced apoptosis, but it abolished TNFα- or TRAIL-induced necroptosis (Fig. 2e–g and Supplementary Fig. 2e). Taken together, these data suggested that MLKL depletion enhances TRAIL-induced apoptosis, but not necroptosis, regardless of RIP3 status.

### RIP3-dependent MLKL phosphorylation is not required for TRAIL-mediated apoptosis

**Fig. 3:**
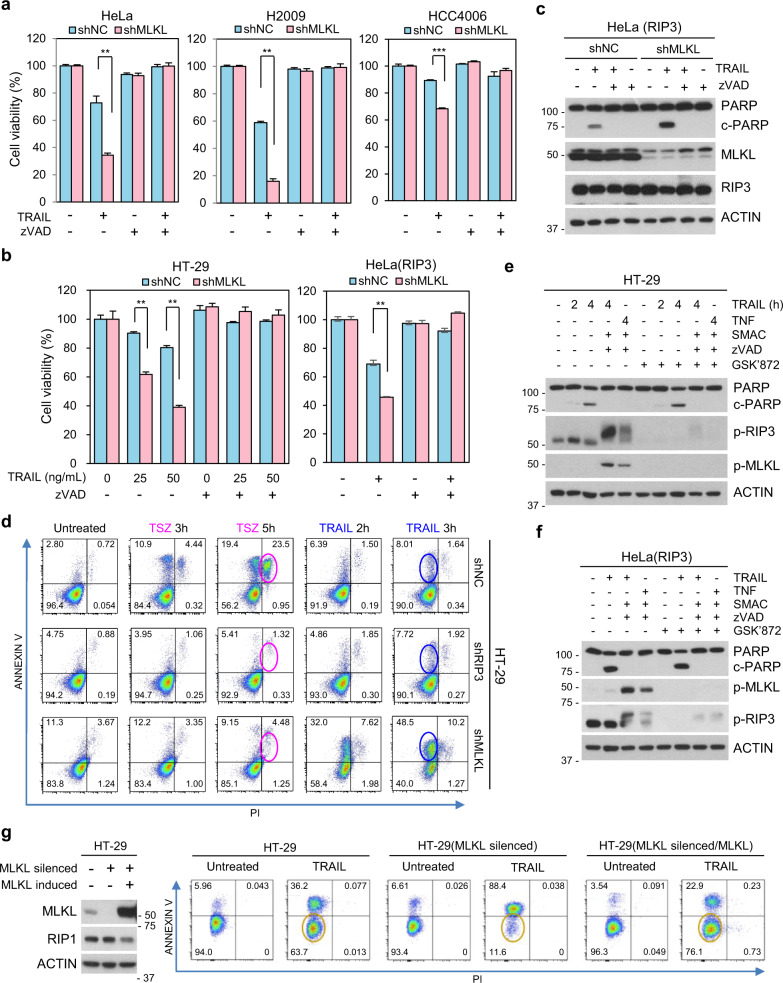
MLKL depletion-mediated sensitization was blocked by caspase inhibition. **a** HeLa, H2009, and HCC4006 cells expressing MLKL shRNA, or a non-silencing control were pretreated with zVAD (20 μM) for 1 h and then treated with TRAIL for 24 h. Cell viability was analyzed by MTT assay. The results are presented as means ± SEM. *P < 0.05, **P < 0.01, ***P < 0.001. **b** HT-29 and RIP3-expressing HeLa cells expressing MLKL shRNA, or a non-silencing control were pretreated with zVAD for 1 h and then treated with TRAIL for 48 or 12 h. Cell viability was analyzed by MTT assay. The results are presented as means ± SEM. *P < 0.05, **P < 0.01, ***P < 0.001. **c** RIP3-expressing HeLa cells expressing MLKL shRNA, or a non-silencing control were pretreated with zVAD for 1 h and then treated with TRAIL (5 ng/ml) for 6 h. The cells lysates were analyzed by western blotting. **d** PI/annexin V staining to distinguish apoptosis and necroptosis in HT-29 shNC, shRIP3 and shMLKL cells. Cells were pretreated with SMAC mimetic + zVAD for 1 h and then treated with TNF (30 ng/ml) or treated with TRAIL (100 ng/ml) for indicated time points and stained with PI/annexin V then analyzed by FACS. **e**, **f** HT-29 (**e**) and RIP3-expressing HeLa (**f**) cells were pretreated with GSK’872 for 1 h and then treated with TRAIL (100 ng/ml or 5 ng/ml) and cell lysates were analyzed by western blotting. **g** Cell death analysis by PI/annexin V staining in HT-29 and MLKL-siRNA-silenced HT-29 cells inducibly expressing MLKL. these cells were pretreated with tamoxifen (1 μM) for 10 h and then treated with TRAIL (100 ng/ml) for 8 h.

We next verified that TRAIL-dependent death was acting by caspase-dependent processes^23^ in our cells. As shown in Fig. 3a–c, various cancer cells (either in the presence or absence of MLKL knockdown) completely lost their sensitivity to TRAIL when treated with the pan-caspase inhibitor zVAD; lack of necroptotic cell death is consistent with the fact that these cells lack RIP3 expression. However, even in cells with RIP3 expression, zVAD completely abolished TRAIL-induced cell death and PARP cleavage (Fig. 3b, c) indicating that TRAIL-induced cytotoxicity was purely apoptotic when cells were treated with the ligand alone (this in contrast to when cells were treated with a SMAC mimetic in combination with TRAIL, see Fig. 2d). To further delineate between apoptosis and necroptosis, we repeated experiments using a combination of PI and annexin V staining, which is often used for such a purpose. As shown in Fig. 3d, MLKL-depleted HT-29 cells had an increased annexin V-positive/ PI negative cell population in response to TRAIL in comparison with control HT-29 cells and RIP3 knockdown HT-29 cells. All three populations lacked many annexin V-positive/PI positive cells (necroptotic/late apoptotic cells) in response to TRAIL, however, only control HT-29 cells had increased PI/annexin V-double positive cells in response to the treatment with TNFα, SMAC mimetic and zVAD, which is used to induce necroptosis (Fig. 3d). RIP3 expressing HeLa cells acted similarly to HT-29 cells; TRAIL-treated HeLa(RIP3) had annexin V-positive/ PI negative cells indicating apoptosis, but always lacked the PI/annexin V-double positive cells indicative of necroptosis unless SMAC mimetic was added additionally to the treatment conditions (Supplementary Fig. 3a, b). We, therefore, conclude that MLKL contributes to apoptosis, as evidenced by the following: (1) MLKL depletion increases TRAIL-upregulated cleaved-PARP/cleaved caspase 3 levels (2) MLKL depletion remarkably increases apoptosis in response to TRAIL, and (3) MLKL depletion-mediated sensitization of TRAIL-cytotoxicity is inhibited by zVAD treatment.

Since our data suggested that MLKL-depleted cells enhanced TRAIL-induced apoptosis, we further validated whether RIP3 kinase activity is required for such sensitivity. As expected, the RIP3 kinase inhibitor GSK’872 had no effect on RIP3-expressing HeLa cells in response TRAIL alone or TRAIL plus SMAC mimetic (apoptotic conditions) but did inhibit TRAIL- or TNF-induced necroptosis induced by these ligands in the presence of SMAC mimetic and zVAD (Supplementary Fig. 3c). GSK’872 properly inhibited the appearance of phospho-RIP3 and phospho-MLKL under necroptotic conditions in HT-29 and HeLa (RIP3) cells but did not inhibit PARP cleavage under apoptotic conditions (Fig. 3e, f).

To exclude off-target effects of RNA interference, we used an inducible system to restore MLKL in MLKL knockdown cells and observed effects on TRAIL cytotoxicity. In these cells, 5-hydroxytamoxifen treatment restores MLKL in the MLKL silenced cells (Supplementary Fig. 3d). As shown in Fig. 3g, MLKL depletion sensitized TRAIL-induced cell death but 5-hydroxytamoxifen treatment restored MLKL expression and, at the same time, decreased sensitivity to TRAIL. Consistent with previous data in MLKL overexpressing HeLa cells, high amount of MLKL expression in the MLKL inducible HT-29 cells did not promote the cell death rate beyond that of the parental HT-29 cells (Fig. 3g and see the Supplementary Fig. 1c).

### Depletion of MLKL caused defect on receptor-ligand endosomal trafficking

**Fig. 4:**
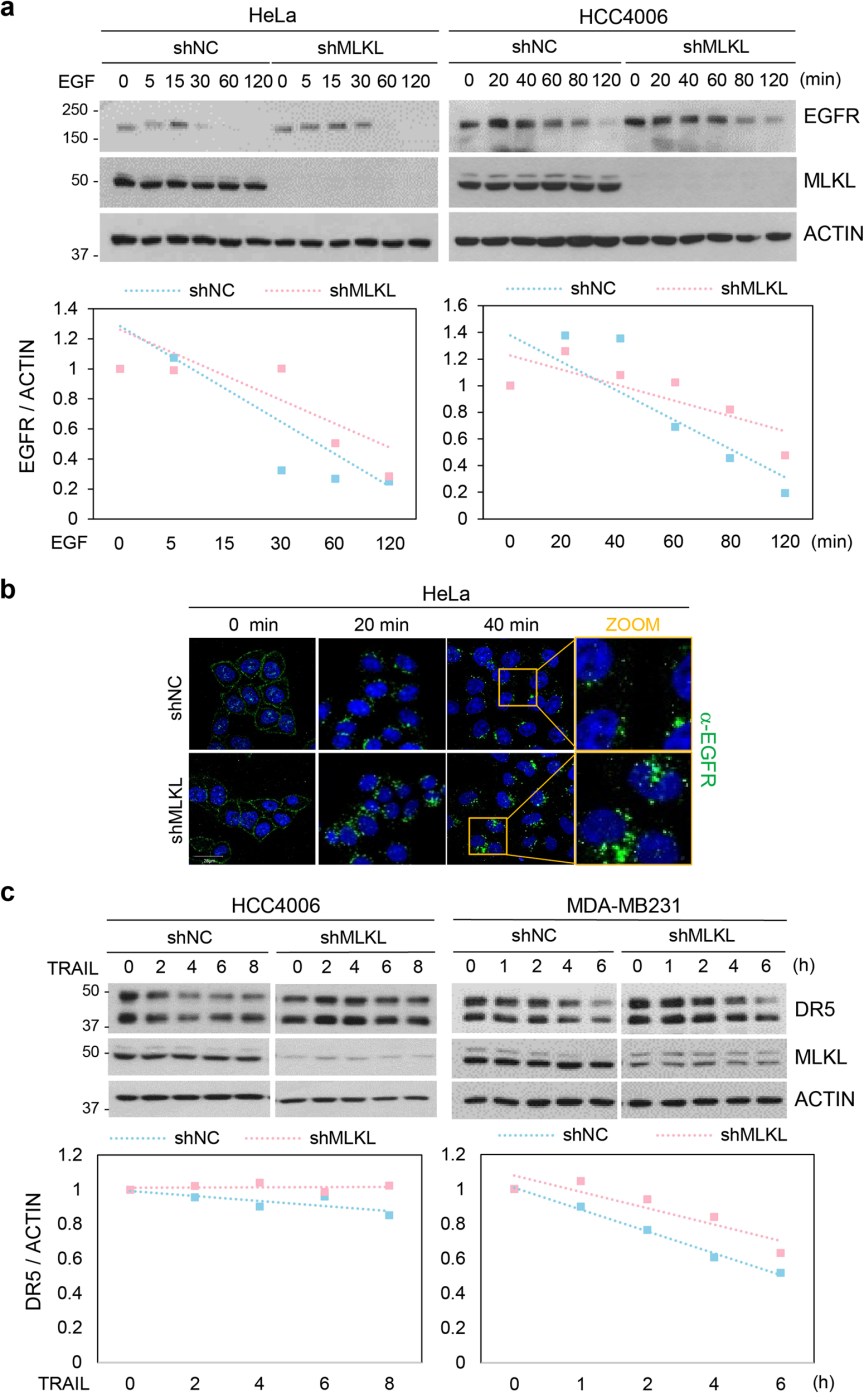
Depletion of MLKL delays receptor degradation. **a** HeLa and HCC4006 cells stably expressing MLKL shRNA or a non-silencing control were treated with EGF (100 ng/ml) in time-dependent manner. EGFR status was analyzed by western blotting (upper panel) and performed using ImageJ quantification software to measure the relative band intensity (bottom panel). **b** HeLa cells stably expressing MLKL shRNA or a non-silencing control were treated with EGF (100 ng/ml) in a time-dependent manner. Cells were stained with EGFR antibody and analyzed by confocal fluorescence microscopy (green: EGFR, blue: DAPI). **c** HCC4006 and MDA-MB231 cells stably expressing MLKL shRNA or non-silencing control were treated with TRAIL (50 ng/ml) in time-dependent manner. Death receptor 5 (DR5) status was analyzed by western blotting (upper panel) and performed using ImageJ quantification software to measure the relative band intensity (bottom panel).

Endocytosis is characterized by internalization of molecules from the cell surface into internal membrane compartments, where receptors enter the early endosome where they are sorted, and they are either recycled back to the plasma membrane via recycling endosomes or are degraded in the late endosome or lysosomes^24^. Ligand internalization is a receptor-mediated endocytic process in which the cell will only take in an extracellular molecule if it binds to its specific receptor. As mentioned previously, internalization of the TRAIL receptor occurs by a clathrin-dependent mechanism and has been shown to attenuate its apoptotic signaling^20^. We sought to investigate the mechanism by which MLKL silencing increases TRAIL-cytotoxicity. We first sought to confirm what others have reported about the effect MLKL depletion on the cellular response to epidermal growth factor (EGF)^9^. Treating of cells with EGF leads to the rapid downregulation of the cell-surface EGFR and by blocking cell-surface EGFR activation and EGFR recycling back to the cell surface, internalized activated receptors have been shown to signal from endosomes and to promote cell survival. EGFR endocytosis decreases cell viability leading to the attenuation of downstream signaling of EGFR for the activation of molecules such as AKT in the ERK1/2 pathway for cell survival and proliferation^25^. Consistent with the literature, knockdown of MLKL resulted in marked slowdown of the intracellular degradation of EGFR as shown by western blotting (Fig. 4a) and immunofluorescence staining (Fig. 4b). Furthermore, the slowdown of the intracellular degradation of EGFR by MLKL silencing resulted in an increase of several EGF-induced signaling activities (Supplementary Fig. 4a, b). We, therefore, confirm that MLKL silencing affects the EGFR endocytosis/endosomal trafficking as reported.

To determine whether the mechanism accounting for EGFR endocytosis/endosomal trafficking also affects TRAIL, we examined the effects of MLKL depletion on the TRAIL receptor, DR5. Like EGF, we observed a slowdown of degradation of DR5 upon MLKL silencing in both MDA-MB231 and HCC4006 cells (Fig. 4c), and this effect increased with more efficient knockdown. This suggests that depletion of MLKL exerts a general effect on receptor internalization/endosomal trafficking and subsequent degradation. As sensitization of TRAIL-induced cell death is associated with increased expression of death receptor^26^, a defect of internalization and/or trafficking of the TRAIL receptor in the absence of ligand might result in the enhancement of cell death. We, therefore, investigated whether the depletion of MLKL altered the protein or mRNA expression of DR5 and confirmed that there was no general effect in the absence of ligand (Supplementary Fig. 4c–e).

### Enhanced death signal due to the defect of MLKL-mediated endosomal trafficking

**Fig. 5:**
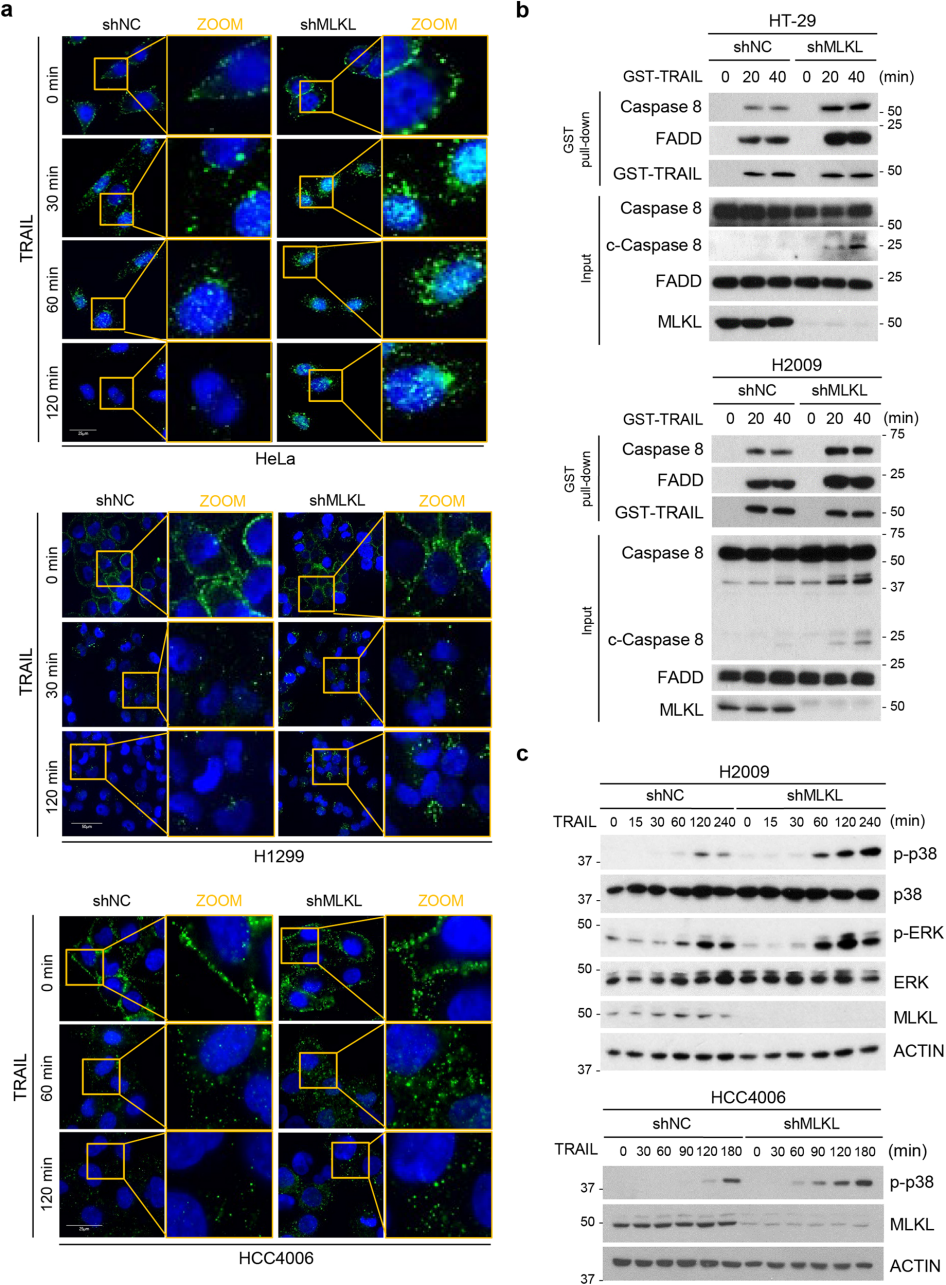
TRAIL-induced cell death and signaling enhances by blocking endocytosis. **a** HeLa, H1299, and HCC4006 cells stably expressing MLKL shRNA or a non-silencing control were treated with GST-TRAIL in a time-dependent manner. Cells were stained with GST antibody and analyzed by confocal fluorescence microscopy (green: GST, blue: DAPI). **b** HT-29 and H2009 cells stably expressing MLKL shRNA or non-silencing control were treated GST-TRAIL for indicated time points and GST-TRAIL bound protein complexes were pulled down by glutathione-agarose beads and analyzed by western blotting. **c** H2009 and HCC4006 cells stably expressing MLKL shRNA or non-silencing control were treated GST-TRAIL (25 ng/ml or 50 ng/ml) in a time-dependent manner. Cell lysates were analyzed by western blotting.

Our data clearly imply that the link between MLKL-mediated endosomal trafficking and TRAIL receptor endocytosis/trafficking affects TRAIL-induced cytotoxicity. Importantly, immunofluorescence staining of the intracellular fate of the TRAIL in cells showed that it was initially present at the cell surface in similar patterns in both MLKL-expressing cells and MLKL-depleted cells (Fig. 5a). However, at later time points, degradation of intracellular TRAIL was delayed/reduced in MLKL-depleted cells compared to MLKL-expressing cells (Fig. 5a). The increased amount of intracellular TRAIL implies that depletion of MLKL interferes the intracellular trafficking of TRAIL-DR5 to degradative compartments.

To further elucidate the effect of MLKL-depletion on endosomal trafficking mediated sensitization of TRAIL-cytotoxicity, we examined whether MLKL depletion can facilitate the association/stabilization of components for DISC complex. A GST-pull down assay showed that the FADD and caspase-8 recruitment to TRAIL receptor were much higher in MLKL-depleted HT-29 and H2009 cells (Fig. 5b) compared with MLKL-expressing control cell line. In addition, the activated form of caspase-8 was increased in MLKL depleted cells compared with control cells. These results demonstrate that MLKL depletion enhances DISC (death-inducing signaling complex) formation and contributes to increased sensitivity to TRAIL-induced apoptosis. Consistent with enhanced DISC formation, TRAIL-induced downstream signals such as p38 and ERK MAPK activation were also enhanced in MLKL depleted cells (Fig. 5c). Since endosomes are often targeted to the lysosome for degradation, we tested whether lysosomal proteases were responsible for the eventual degradation of DR5. Treatment of TRAIL-stimulated cells with lysosomal inhibitors, pepstatin A plus E64D, arrested DR5 decreases suggesting that the intracellular degradation of TRAIL–DR complex is mediated by lysosomal proteases downstream of endosomal trafficking (Supplementary Fig. 4f).

### Endosomal trafficking is associated with MLKL-mediated sensitization to TRAIL-cytotoxicity

**Fig. 6:**
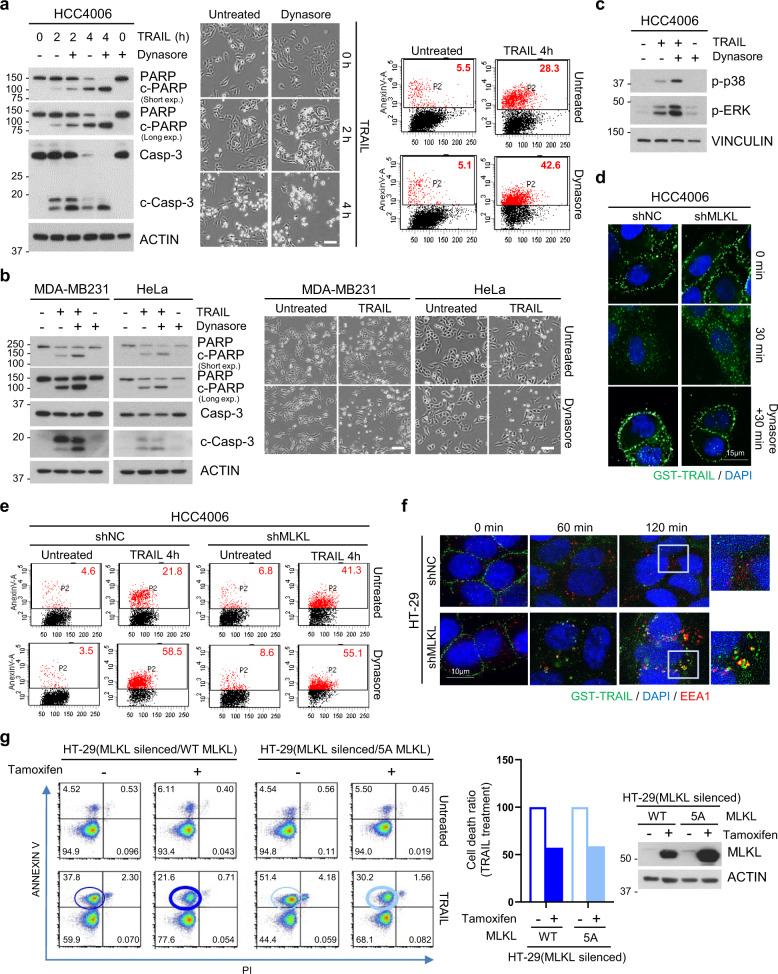
Endosomal trafficking is associated with TRAIL-cytotoxicity. **a** HCC4006 cells were pretreated with dynasore (80 μM) for 1 h and then treated with TRAIL (50 ng/ml) in time-dependent manner. Cell lysates were analyzed by western blotting (left panel) and cell viability was analyzed by phase-contrast microscopy (middle panel) or FACS analysis after Annexin V staining (right panel). Scale bars, 100 μm. **b** MDA-MB231 and HeLa cells were pretreated with dynasore (80 μM) for 1 h and then treated with TRAIL (5 ng/ml or 2.5 ng/ml). Cell lysates were analyzed by western blotting (left panel) and cell viability was analyzed by phase-contrast microscopy (right panel). Scale bars, 100 μm. **c** HCC4006 cells were pretreated with dynasore (80 μM) for 1 h and then treated with TRAIL (50 ng/ml) for 4 h. Cell lysates were analyzed by western blotting. **d** HCC4006 cells expressing MLKL shRNA, or non-silencing control were pretreated with dynasore (80 μM) for 1 h and then treated with GST-TRAIL for 30 min. Cells were stained with GST antibody and analyzed by confocal fluorescence microscopy (green: GST, blue: DAPI). **e** HCC4006 cells expressing MLKL shRNA, or non-silencing control were pretreated with dynasore (80 μM) for 1 h and then treated with TRAIL (50 ng/ml) for 4 h. Cell viability was analyzed by FACS analysis after Annexin V staining. **f** HT-29 cells expressing MLKL shRNA, or non-silencing control were treated with GST-TRAIL for indicated time points. Cells were co-stained with GST and EEA1 antibodies and analyzed by confocal fluorescence microscopy (green: GST, blue: DAPI, red: EEA1). **g** Cell death analysis by PI/annexin V staining in MLKL-siRNA-silenced HT-29 cells inducibly expressing WT MLKL and 5A mutant MLKL. These cells were pretreated with tamoxifen (1 μM) for 10 h and then treated with TRAIL (100 ng/ml) for 8 h.

Since our data suggest a role for MLKL in maintaining the endosomal trafficking, we examined whether directly blocking receptor internalization similarly increases TRAIL-induced cell death. Dynamins are master regulators of clathrin-mediated endocytosis (CME) and control the earlier rate-limiting steps of clathrin-coated vesicle formation^27–29^. Using the dynamin inhibitor, dynasore, we prevented TRAIL-induced DR5 internalization in different cell types. As expected, TRAIL-induced cell death was boosted in the presence of dynasore indicating that dynamin-dependent endocytosis of TRAIL–DR complexes suppress apoptotic signaling (Fig. 6a, b and Supplementary Fig. 5a). Similar to MLKL depletion, dynasore treatment also resulted in increased TRAIL-induced signaling activities, such as phosphorylation of ERK and p38 (Fig. 6c and Supplementary Fig. 5b), indicating that prolonged ligand-receptor signaling by blockage of endocytosis enhances downstream effects. In the presence of dynasore, TRAIL-DR5 complex internalization and degradation was delayed, and MLKL depletion had no further effect on TRAIL-induced cell death (Fig. 6d, e), suggesting that elimination of receptor internalization and trafficking abolished the MLKL-depletion mediated mechanism of sensitization to TRAIL-cytotoxicity. Therefore, endosomal trafficking appears to be necessary for MLKL depletion-induced increases in TRAIL cytotoxicity. Further immunocytochemical analysis of the intracellular fate of the TRAIL-DR5 complex in cells showed that it was taken up into the cells at about the same rate in both MLKL-expressing and MLKL-depleted cells, however, after internalization, TRAIL accumulated in early endosomes in MLKL-depleted cells as shown by increased localization with EEA1 (Fig. 6f and Supplementary Fig. 6a, b). The observed effects of MLKL depletion on the rates of intracellular degradation of TRAIL and its receptor, coupled with these observations suggest that the role of MLKL is maintain the endosomal trafficking downstream of endocytosis.

Phosphorylation of MLKL by RIP3 activation can contribute to endosomal trafficking and EV generation at an enhanced grade but the constitutive contribution of MLKL to these functions does not depend on RIP3^9^. So far, our data suggest that MLKL depletion-mediated TRAIL sensitivity is not related with RIP3 status and the enhanced death signal is due to the defect of MLKL-mediated endosomal trafficking; therefore, the conformational change of MLKL that is associated with necroptosis may not be absolutely required for its role in intracellular trafficking of TRAIL–DR complex. To address whether this was the case, we examined whether an MLKL membrane localization mutant (5A) had the ability to rescue the TRAIL sensitization observed upon MLKL knockdown. The mutant protein has mutations in five basic residues in the N-terminal α helical bundle region which are required for MLKL functions of membrane binding, necroptosis, and the generation of extracellular vesicles (EVs)^9^, which are mediated by MLKL phosphorylation by RIP3 and possibly other kinases. When MLKL silenced cells were reconstituted with 5A mutant MLKL expression, it repressed the cell death sensitization due to MLKL depletion just as the WT MLKL protein did (Fig. 6g), however, the mutant was unable to rescue TRAIL-induced necroptosis (Supplementary Fig. 6c). This suggests that the normal structural elements of MLKL that are necessary for necroptosis and EV generation are not required for MLKL dependent repression of cell death that our previous data has attributed to the regulation of endosomal trafficking. This is consistent with the previous data that RIP3, which phosphorylates MLKL, is not required for its effects on apoptosis, and is consistent with the lack of RIP3 and MLKL phosphorylation upon treatment with TRAIL ligand alone (Supplementary Fig. 6d).

While we previously showed that TRAIL-DR5 complexes were initially present at the cell surface in similar patterns in both MLKL-expressing cells and MLKL-depleted cells and was initially taken up into the cells at about the same rate at early points (see Fig. 6f and Supplementary Fig. 6a, b), it is possible that MLKL could also influence endosomal trafficking at later points by regulating caspase activity to prevent further endocytosis. It has been known that DR5 activation promotes rapid and specific cleavage of proteins such as adaptor protein 2 (AP2) which link clathrin to cytoplasmic determinants of endocytic cargo during the formation of clathrin-coated pits^15^. To understand whether MLKL depletion-mediated TRAIL receptor degradation works with caspase to regulate clathrin-mediated endocytosis, we detected whether AP2 was more substantially cleaved in MLKL depleted cells. Concomitant with MLKL depletion-mediated increased PARP cleavage, in the MLKL deficient cells showed cleavage of AP2 suggesting that TRAIL–DR complex formation promotes potent cleavage of protein involved in clathrin-dependent endocytosis (Supplementary Fig. 6e–g). Pretreatment with zVAD prevented cleavage of PARP and AP2 (Supplementary Fig. 6h), indicating that resultant caspase activity during TRAIL would likely disproportionally affect endocytosis of DISC complex, which previously showed much higher caspase-8 recruitment to TRAIL receptor in MLKL depleted cells (see Fig. 5b).

## Discussion

MLKL is a key effector of necroptosis and upon activation by phosphorylated RIP1, RIP3 will recruit and phosphorylate MLKL to execute necroptosis^30^. During this process, MLKL forms an oligomer and translocates to the plasma membrane to cause membrane rupture. In addition to RIP3-dependent activation of MLKL, MLKL also functions in extracellular vesicle generation via a RIP3-independent manner. The observed effects of MLKL depletion on the rates of intracellular degradation of EGF and its receptor pointed to a constitutive role for MLKL in maintaining the endosomal trafficking^9^.

Due to fact that the signaling from activated TRAIL receptors occurs mostly from the plasma membrane, endocytosis is thought to initiate the termination of the signal, and trafficking defects are associated with enhanced ligand-receptor signals. In this study, we found that depletion of MLKL accelerates TRAIL-induced cancer cell apoptosis, and that enhancement of such cell death by MLKL depletion occurs via a RIP3-independent manner. Since the expression of RIP3 is lost/reduced in a large number of cancer cells/tumor tissues due to DNA methylation of the *RIPK3* genomic sequence^22,31,32^, it is fortunate that this is the case, since a therapeutic reduction of MLKL in cancer cells may still mediate increased cancer cell death in these cancers, making MLKL inhibition a potential therapeutic strategy for cancer treatment in the presence of TRAIL pathway activators.

Depletion of MLKL caused apparent defects in receptor-ligand endosomal trafficking of TRAIL and resulted in prolonged death signals due to a TRAIL–DR trafficking defect. Trafficking defects of TRAIL–DR were shown in depletion of MLKL, as evidenced by the following: (1) TRAIL degradation and the typical post-signaling reduction of plasma membrane-associated TRAIL was delayed in MLKL-depleted cells (2) prolonged/enhanced intracellular signals such as p-ERK and p-p38 occurred in MLKL-depleted cells, (3) a slowdown of degradation of DR5 in response to TRAIL by occurred upon MLKL silencing, and (4) immunocytochemical analysis of the intracellular fate of the TRAIL-DR5 complex in cells showed that it was taken up into the cells at about the same rate in both MLKL-expressing and MLKL-depleted cells, however, after internalization, TRAIL accumulated in early endosomes in MLKL-depleted cells as shown by increased localization with EEA1. Interestingly, the function of MLKL in endosomal trafficking does not require the normal N-terminal structural elements of MLKL that are necessary for the conformational change of MLKL that is associated with necroptosis and extracellular vesicle generation, suggesting a largely different mechanistic set of interactions in regulating endosomal trafficking.

As noted generally, TRAIL’s ability to induce apoptosis in cancer cells, led to the clinical development of several agonists for TRAIL-TRAIL receptors. However, to date none of these TRAIL receptor agonists has produced significant clinical benefits in large numbers of cancer patients in clinical trials. One of reason for clinical failure is that lack of suitable biomarkers to identify patients who are more likely to respond to a TRAIL receptor agonist-comprising therapy^33^. In this point, we propose that patients who have a relatively low level of MLKL in tumor tissues are more likely to respond to TRAIL receptor agonists due to the perturbation in endosomal trafficking of the TRAIL receptor agonist. Abnormal expression of MLKL has been detected in many kinds of tumors, such as colon cancer, ovarian cancer, and gastric cancer^34–36^ and recent studies also have revealed that MLKL could serve as a potential prognostic biomarker for patients with cancer^37–39^. In these studies, the authors revealed that decreased expression of MLKL was significantly associated with poor overall survival in cancer patients suggesting a prognostic and clinicopathological significance of expression level of MLKL in cancer patients. Therefore, we propose that, in view of their poor overall survival cancer patients who have decreased expression level of MLKL in tumor tissues may potentially receive a clinical benefit from TRAIL receptor agonist therapy, as these patients’ cancers may be more potentially responsive to TRAIL therapy.

## Supplementary information

Supplementary Figure Legends

Supplementary Figure 1

Supplementary Figure 2

Supplementary Figure 3

Supplementary Figure 4

Supplementary Figure 5

Supplementary Figure 6
