# Supplementary material for: Reduction in MLKL-mediated endosomal trafficking enhances the TRAIL-DR4/5 signal to increase cancer cell death
Source: Cell Death Dis. 2020 Sep 11;11(9):744. doi: 10.1038/s41419-020-02941-9 (PMC7486371; doi:10.1038/s41419-020-02941-9)
Supplement: Supplementary file 1 — Supplementary Figure Legends [file 41419_2020_2941_MOESM1_ESM.docx]

**Supplementary Figure Legends**

**Supplementary Figure 1. Depletion of MLKL accelerates TRAIL-induced apoptosis.**

(A) HeLa cells expressing MLKL shRNA, or a non-silencing control were treated with varying doses of TRAIL for 12 hours and cell viability was analyzed by MTT assay (respond to Main Fig. 1a).

(B) HeLa, HCC4006 and H2009 cells stably expressing MLKL shRNA or non-silencing control were treated with TRAIL in a time-dependent manner. Cell lysates were analyzed by western blotting.

(C) MLKL-overexpressing stable cells were treated with TRAIL (30 ng/ml) for indicated time points and cell lysates were analyzed by western blotting (upper panel). Cells were also treated with various concentration of TRAIL for 18 hours and cell viability was analyzed by MTT assay (bottom panel).

**Supplementary Figure 2. MLKL dependency in response to the chemotherapeutic agent and TNF-induced necroptosis.**

(A) HT-29 and H2009 cells expressing MLKL shRNA, or a non-silencing control were pretreated with SMAC mimeticfor 1 hour and then treated TNF and TRAIL for 24 hours. Cell viability was analyzed by MTT assay. The results are presented as means ± SEM. ***P* < 0.01, ****P* < 0.001.

(B) HT-29 cells stably expressing MLKL shRNA, RIP3 shRNA or a non-silencing control were treated with indicated concentration for 48 hours and cell viability was analyzed by MTT assay. The results are presented as means ± SEM. ***P* < 0.01, ****P* < 0.001.

(C) HT-29 cells stably expressing MLKL shRNA or a non-silencing control were treated with etoposide for 48 hours. Cell viability was analyzed by MTT assay. The results are presented as means ± SEM. **P* < 0.05, ***P* < 0.01.

(D) HeLa cells stably expressing MLKL shRNA or a non-silencing control were treated with etoposide for 24 hours (upper panel). Cells were also treated etoposide (50 μM) for the indicated time points and cell lysates were analyzed by western blotting (bottom panel).

(E) RIP3-expressing HeLa cells expressing MLKL shRNA, or a non-silencing control were treated with TRAIL indicated concentration for 12 hours and cell viability was analyzed by MTT assay. The results are presented as means ± SEM. ***P* < 0.01, ****P* < 0.001.

**Supplementary Figure 3. MLKL depletion increases apoptosis in response to TRAIL and RIP3 kinase activity is required for such sensitivity.**

(A) RIP3-expressing HeLa cells were treated with indicated conditions and cell viability was analyzed by PI/annexin V staining.

(B) HeLa and RIP3-expressing HeLa cells were treated with indicated conditions and cell viability was analyzed by PI/annexin V staining.

(C) RIP3-expressing HeLa cells were treated with indicated concentration for 24 hours in the absence or presence of GSK’872 pretreatment for 1 hour and cell viability was analyzed by MTT assay. The results are presented as means ± SEM. ****P* < 0.001.

(D) Western blotting of the expressed MLKL in MLKL-siRNA-silenced HT-29 cells inducibly expressing MLKL.

**Supplementary Figure 4. MLKL silencing affects the EGFR endocytosis but depletion of MLKL had no effect on alteration of the protein or mRNA expression of DR5.**

(A) H2009 cells stably expressing MLKL shRNA or a non-silencing control were treated with EGF (100 ng/ml) in time-dependent manner. Cell lysates were analyzed by western blotting.

(B) HeLa and HepG2 cells stably expressing MLKL shRNA or non-silencing control were treated with EGF (100 ng/ml) in time-dependent manner.

(C) Death Receptor 5 expression pattern upon MLKL depletion. Cell lysates were analyzed by FACS analysis. (H1299 - Red: Unstain, Sky blue: DR5 stain / HT-29 - Sky blue: Unstain, blue: DR5 stain)

(D) Death Receptor 5 mRNA levels in various cancer cells with MLKL depletion.

(E) Death Receptor 5 expression pattern upon MLKL depletion. Cell lysates were analyzed by western blotting.

(F) The effect of arrest of lysosomal degradation by Pepstatin A plus E64D treatment on the amount of DR5.

**Supplementary Figure 5. TRAIL-induced cell death and signaling were boosted in the presence of dynasore.**

(A) HeLa cells were pretreated with dynasore (80 μM) for 1 hour and then treated with TRAIL for 12 hours. Cell viability was analyzed by MTT assay. The results are presented as means ± SEM. **P* < 0.05, ***P* < 0.01, ****P* < 0.001.

(B) MDA-MB231 and HeLa cells were pretreated with dynasore (80 μM) for 1 hour and then treated with TRAIL. Cell lysates were analyzed by western blotting.

**Supplementary Figure 6. MLKL influences endosomal trafficking at later points by regulating caspase activity to prevent further endocytosis.**

(A, B) HCC4006 (A) and HT-29 (B) cells expressing MLKL shRNA, or non-silencing control were treated with GST-TRAIL for indicated time points. Cells were co-stained with GST and EEA1 antibodies and analyzed by confocal fluorescence microscopy (Green: GST, Blue: DAPI, Red: EEA1).

(C) Cell death analysis by MTT assay in MLKL-siRNA-silenced HT-29 cells inducibly expressing WT MLKL and 5A mutant MLKL. These cells were pretreated with tamoxifen (1 μM) for 10 hours and then treated with TRAIL + SMAC + zVAD .

(D) HT-29 cells were treated with indicated conditions and cell lysates were analyzed by western blotting.

(E) HeLa cells stably expressing MLKL shRNA or a non-silencing control were treated with various concentration of TRAIL for 6 hours and cell lysates were analyzed by western blotting.

(F) HT-29 cells stably expressing MLKL shRNA or a non-silencing control were treated with TRAIL (50 ng/ml) for indicated time points and cell lysates were analyzed by western blotting.

(G) Western blotting in MLKL-siRNA-silenced HT-29 cells inducibly expressing WT MLKL and 5A mutant MLKL.

(H) RIP3-expressing HeLa cells expressing MLKL shRNA, or a non-silencing control were pretreated with zVAD for 1 hour and then treated with TRAIL (5 ng/ml) for 6 hours. The cells lysates were analyzed by western blotting.
