# Supplementary figures and images for: Reduction in MLKL-mediated endosomal trafficking enhances the TRAIL-DR4/5 signal to increase cancer cell death
Source: Cell Death Dis. 2020 Sep 11;11(9):744. doi: 10.1038/s41419-020-02941-9 (PMC7486371; doi:10.1038/s41419-020-02941-9)

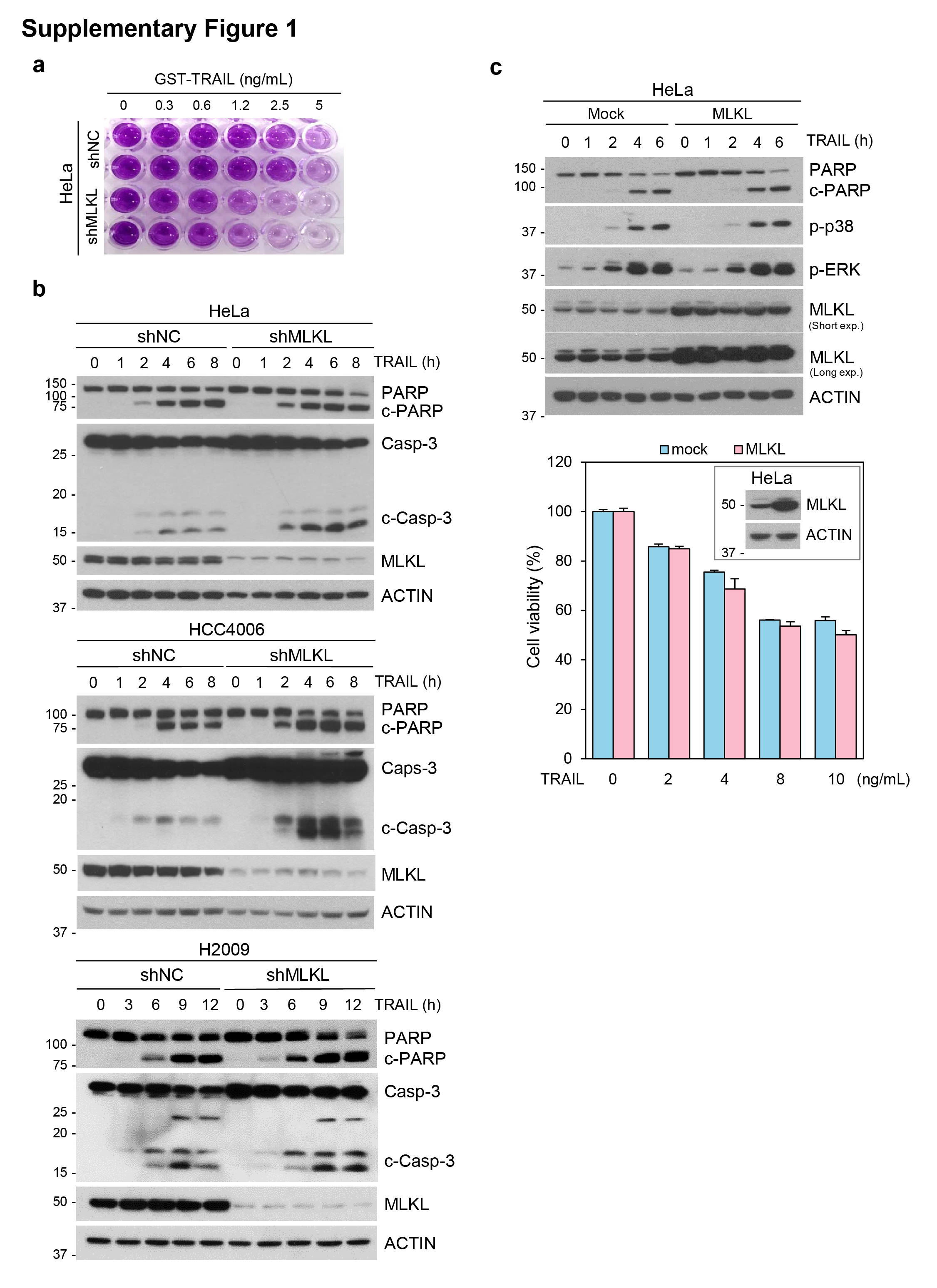

Supplement: Supplementary file 2 — Supplementary Figure 1 [file 41419_2020_2941_MOESM2_ESM.jpg]

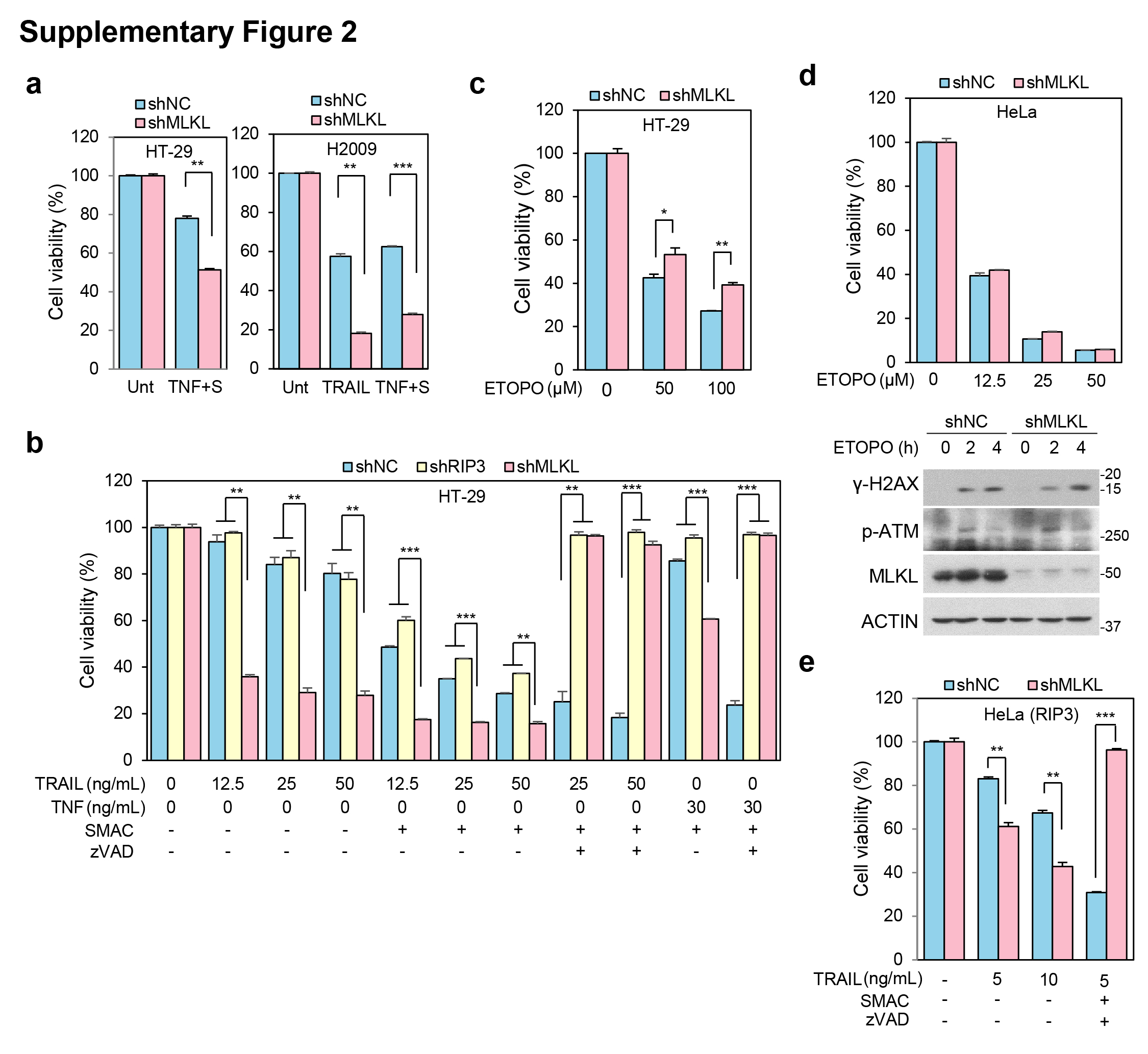

Supplement: Supplementary file 3 — Supplementary Figure 2 [file 41419_2020_2941_MOESM3_ESM.jpg]

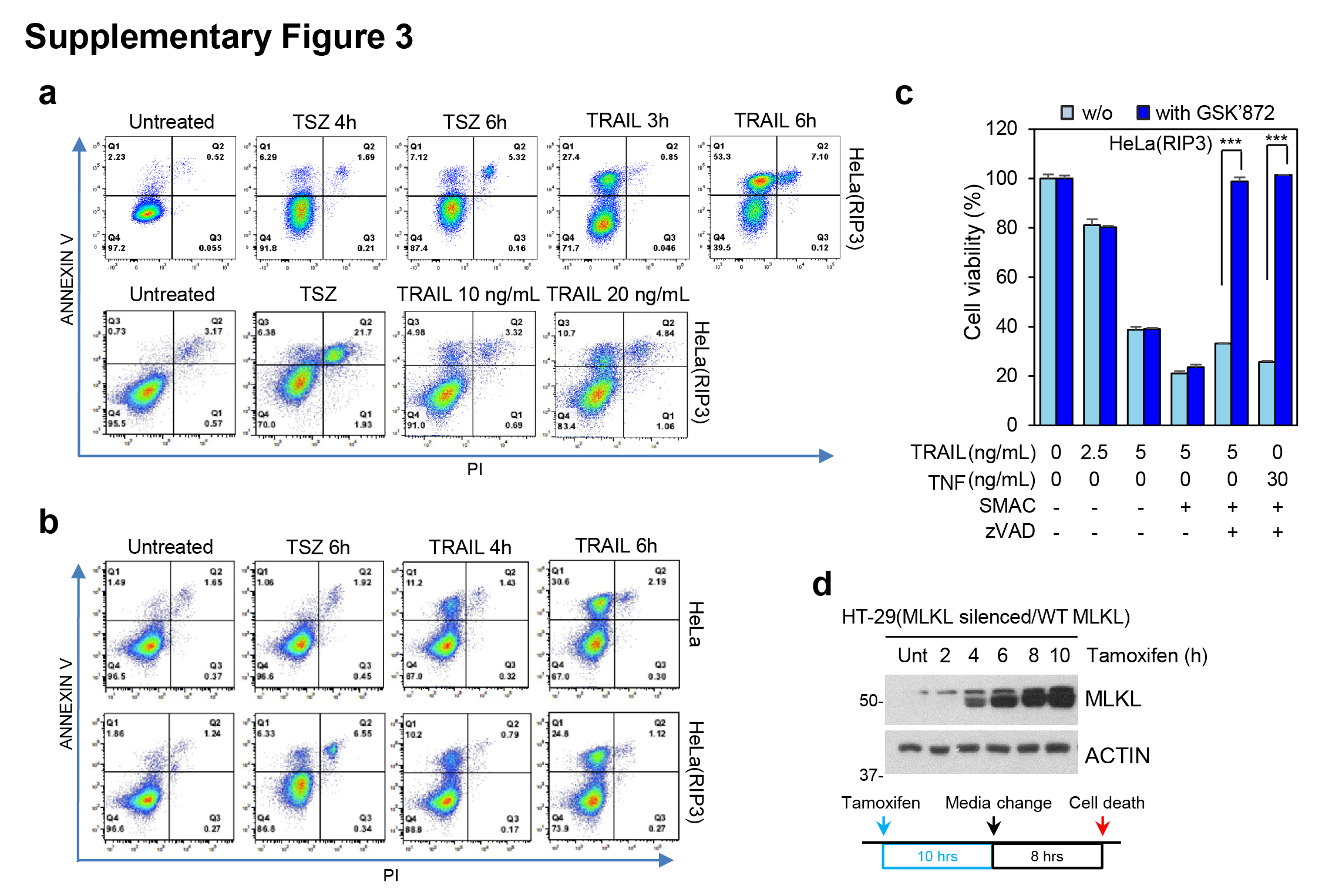

Supplement: Supplementary file 4 — Supplementary Figure 3 [file 41419_2020_2941_MOESM4_ESM.jpg]

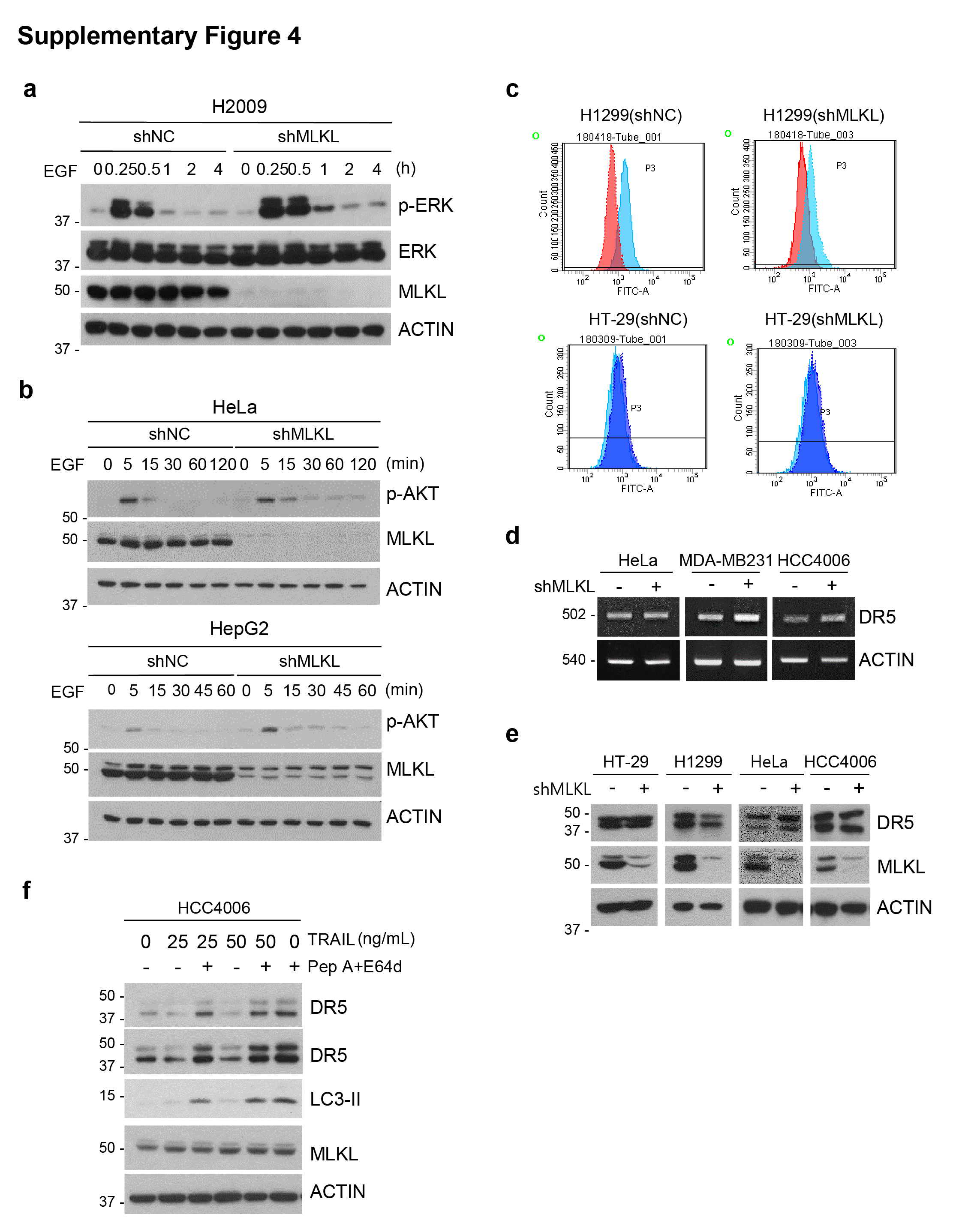

Supplement: Supplementary file 5 — Supplementary Figure 4 [file 41419_2020_2941_MOESM5_ESM.jpg]

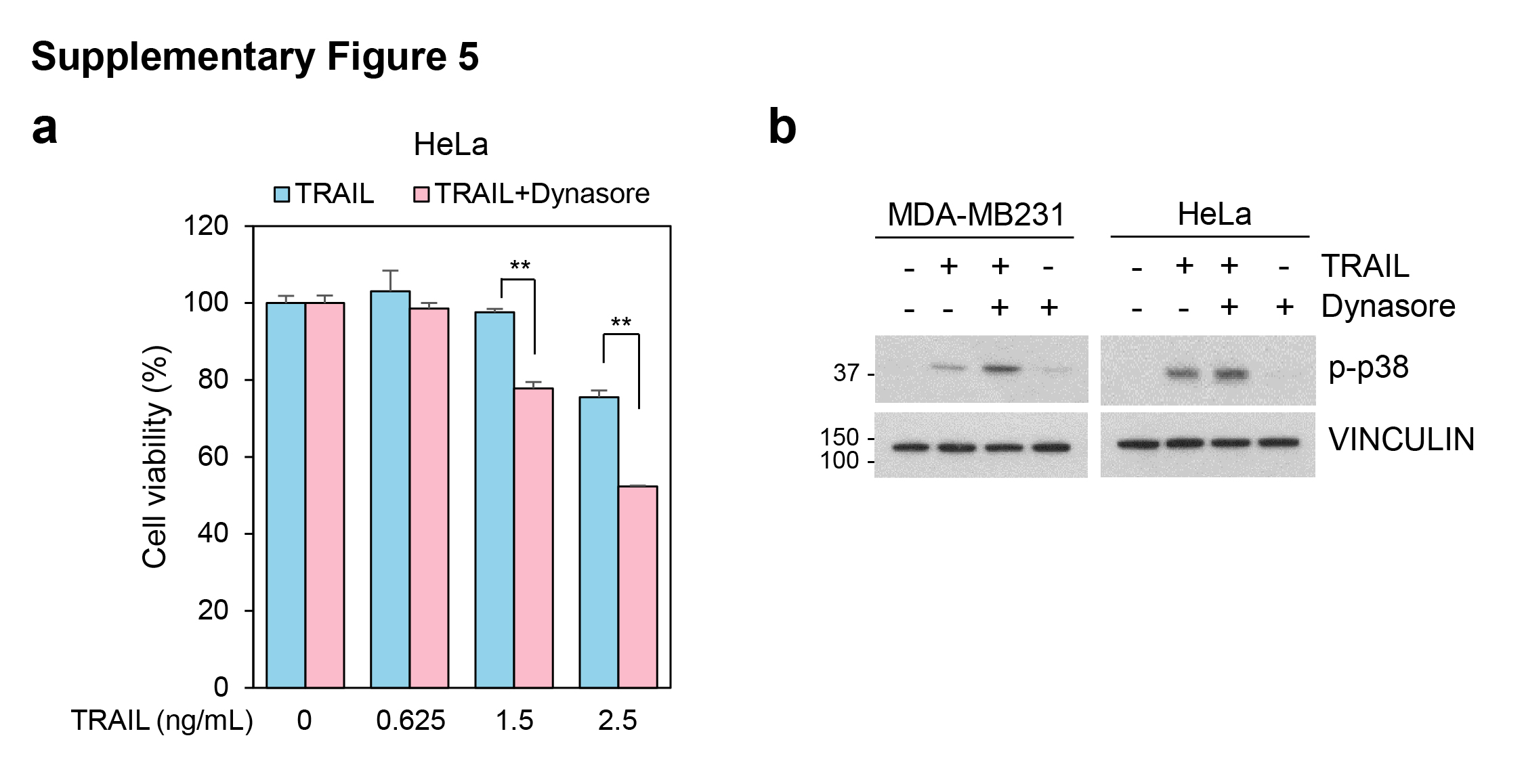

Supplement: Supplementary file 6 — Supplementary Figure 5 [file 41419_2020_2941_MOESM6_ESM.jpg]

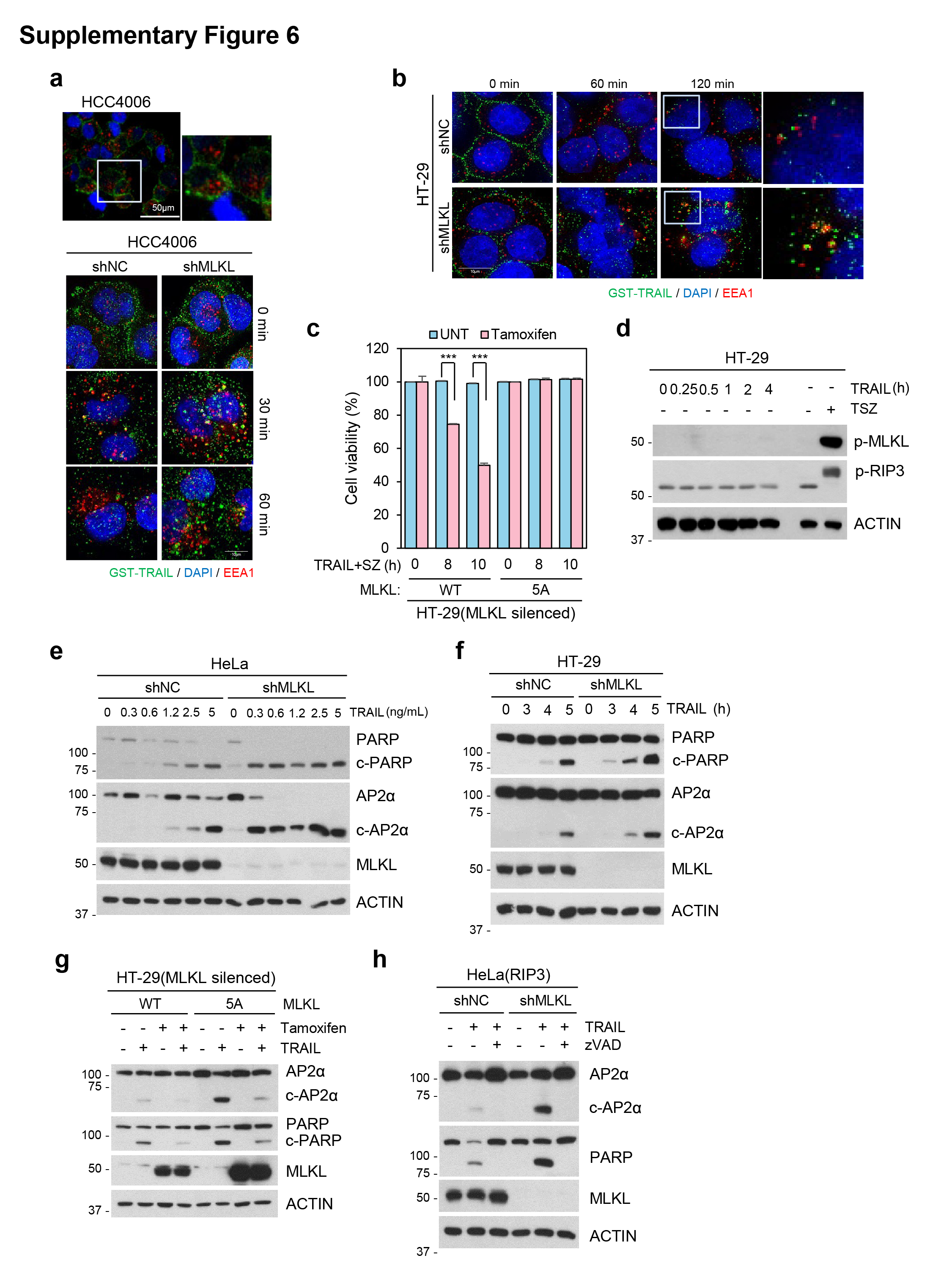

Supplement: Supplementary file 7 — Supplementary Figure 6 [file 41419_2020_2941_MOESM7_ESM.jpg]
